# Impact of Leaf Traits on Temporal Dynamics of Transpired Oxygen Isotope Signatures and Its Impact on Atmospheric Vapor

**DOI:** 10.3389/fpls.2017.00005

**Published:** 2017-01-18

**Authors:** Maren Dubbert, Angelika Kübert, Christiane Werner

**Affiliations:** Ecosystem Physiology, University FreiburgFreiburg, Germany

**Keywords:** transpiration, stable water isotopes, laser spectroscopy, leaf traits, stomatal conductance, leaf water content, isotopic non-steady state

## Abstract

Oxygen isotope signatures of transpiration (δ_*E*_) are powerful tracers of water movement from plant to global scale. However, a mechanistic understanding of how leaf morphological/physiological traits effect δ_*E*_ is missing. A laser spectrometer was coupled to a leaf-level gas-exchange system to measure fluxes and isotopic signatures of plant transpiration under controlled conditions in seven distinct species (*Fagus sylvatica, Pinus sylvestris, Acacia longifolia, Quercus suber, Coffea arabica, Plantago lanceolata, Oxalis triangularis*). We analyzed the role of stomatal conductance (*g*_*s*_) and leaf water content (*W*) on the temporal dynamics of δ_*E*_ following changes in relative humidity (*rH*). Changes in rH were applied from 60 to 30% and from 30 to 60%, which is probably more than covering the maximum step changes occurring under natural conditions. Further, the impact of *g*_*s*_ and *W* on isotopic non-steady state isofluxes was analyzed. Following changes in *rH*, temporal development of δ_*E*_ was well described by a one-pool modeling approach for most species. Isofluxes of δ_*E*_ were dominantly driven by stomatal control on *E*, particularly for the initial period of 30 min following a step change. Hence, the deviation of isofluxes from isotopic steady state can be large, even though plants transpire near to isotopic steady state. Notably, not only transpiration rate and stomatal conductance, but also the leaf traits stomatal density (as a measure of g_max_) and leaf water content are significantly related to the time constant (τ) and non-steady-state isofluxes. This might provide an easy-to-access means of a priori assumptions for the impact of isotopic non-steady-state transpiration in various ecosystems. We discuss the implications of our results from leaf to ecosystem scale.

## Introduction

Plant transpiration is the major flux of water leaving an ecosystem on a global scale (i.e., Jasechko et al., 2013). The isotopic signature of transpired vapor (δ_*E*_) is a powerful tracer of water movement within plants or through ecosystems and can be used to separate net ecosystem water fluxes into the constituent fluxes of plant transpiration and soil evaporation (Yakir and Sternberg, 2000; Dubbert et al., 2014b). In contrast to isotopic signatures of soil evaporation, which are depleted compared to soil water isotopic signatures (δ_*s*_) due to equilibrium and kinetic fractionation associated with phase change and transport of water, it is often assumed that δ_*E*_ is in isotopic steady state, i.e., that the transpired water shows the same isotopic signature as the source water (Brunel et al., 1997; Yepez et al., 2003; Williams et al., 2004; Wang et al., 2013). These strong differences in δ^*18*^ *O* of soil evaporation and plant transpiration provide the basis for partitioning ecosystem ET fluxes. However, recent studies revealed that transpiration is often depleted relative to the isotopic steady state throughout the day (while enriched during the night) in many plant species and ecosystems, which is mainly due to progressive decrease in *rH* toward midday (Lai et al., 2006; Wang et al., 2012; Dubbert et al., 2013, 2014a).

In the past, measurements of water vapor isotopes were time consuming using cold-trap methods (Helliker and Ehleringer, 2002), leading to data-sets with low time resolution (Harwood et al., 1998). δ_*E*_ can also be estimated indirectly by modeling the isotopic signature of leaf water at the evaporating sites of the leaves (δ_*e*_) under the assumption of non-steady-state transpiration (Dongmann et al., 1974). Thereafter, δ_*E*_ can be determined by the Craig and Gordon (1965) equation. Recent developments in laser spectroscopy, however, enable direct measurements of the isotopic composition of atmospheric water vapor (δ_*a*_), evapotranspiration (δ_*ET*_), and its components with high temporal resolution in the field (minute to hourly scale, Werner et al., 2012 and literature therein). Consequently, emerging studies using continuous high-frequency measurements of δ_*E*_ are able to investigate the impact of changing environmental variables (i.e., in relative humidity or leaf temperature). As a general conclusion it seems that periods of stable environmental conditions, allowing for δ_*E*_ to approach isotopic steady state, are mostly too short under natural conditions compared to common leaf water turn-over times (Dubbert et al., 2013, 2014a; Simonin et al., 2013), however studies spanning different environmental conditions and especially across different leaf types are still lacking. Although, some knowledge has been gained on the relation between isotopically non-steady-state leaf water and the consequent non-steady-state effect of transpired vapor (Dubbert et al., 2014a), at present little is known about the effect of species-specific differences in morphological/physiological leaf parameters (stomatal conductance, stomatal density, and water content) on the immediate response of δ_*E*_ to changes in environmental conditions and the time needed for δ_*E*_ under stable environmental conditions to return to an isotopic steady-state.

Moreover, at the ecosystem scale, it is important to understand how distinct leaf traits affect the non-steady-state effects of the transpiration isoflux, which can have a strong forcing on atmospheric vapor. According to isotope theory leaf water content and stomatal conductance, have a direct influence on the time constant (τ) and hence on the development of the isotopic signature of leaf water at the evaporating sites (δ_*e*_) and in consequence the isotopic signature of transpired vapor (δ_*E*_). Stomatal conductance also determines, together with the leaf to air vapor pressure deficit the transpiration rate (*E)*. For example, a strong deviation of the isotopic signature of transpiration from isotopic steady state might not be in concert with low *E*, raising the question of species-specific isoforcing of non-steady-state δ_*E*_ on atmospheric vapor (δ_*a*_). Finally, reliable relationships between relatively easy to measure leaf morphological variables (i.e., leaf water content and stomatal density) and the time constant (τ) and isotopic non-steady-state isoflux might provide first estimates of the impact of isotopic non-steady-state transpiration for modeling the isotope composition of transpiration and leaf water.

To facilitate a better understanding of the relationships between leaf traits and isotopic non-steady-state transitions of δ_*E*_, we used a novel approach, combining a leaf gas-exchange system with a cavity ring-down spectrometer. Under controlled conditions, seven functionally distinct species, including herbs, shrubs, broad leaved, and needle leaved trees were subjected to changes in relative humidity. Our goal was to analyze the relationships between specific leaf traits (i.e., stomatal conductance, stomatal density, leaf water content) on two different levels. We analyzed the relationship between distinct leaf traits and the temporal development of the isotopic signature of transpired vapor. Secondly, we analyzed impacts of leaf traits on the temporal development of the transpirational isoflux. We consider it particularly important to differentiate between the two, as the latter is the actual potential forcing on atmospheric vapor.

## Materials and methods

### Plant material

The experiment was established in the climate chamber facilities at the University of Bayreuth comprising seven species, including two herbaceous species (*Plantago lanceolata* L., *Oxalis triangularis* A. St.- Hil.), two shrubby species (*Acacia longifolia* (Andr.) Willd., *Coffea arabica* L.), and three tree species (*Quercus suber* L., *Fagus sylvatica* L., *Picea abies* (L.) H. Karst.) (see Table 1). This variety of species was chosen to cover different growth forms, leaf types, biomes, and layers of an ecosystem (understory vs. overstory). All plants, except *F. sylvatica* and *P. abies*, were grown in greenhouses. *Fagus sylvatica* and *P. abies* were obtained from a local nursery (Bavaria State Forest Enterprise, Bayreuth). At least 4 weeks before the experiment started, plants were placed in a climate chamber adjusted to an air temperature of 20°C, a relative air humidity (*rH*) of 60%, and a 12 h photoperiod with an average of 400–600 μmol m^−2^ s^−1^ incident at the upper leaf level (depending on the species height). CO_2_-concentrations were not controlled and ranged from 300 to 500 ppm. Plants were kept well watered throughout the entire experiment, were regularly rotated to ensure similar light conditions, and fertilized with Hoagland solution (Peperkorn et al., 2005) and WUXAL Super (Manna, Wilhelm Haug, Ammerbuch-Pfäffingen, Germany). Measurements were conducted starting at least 3 h after start of the light period to ensure that the plants were not a in a transition stage and lasted not longer than 4 h. Isotopic variation of irrigation water was minimized using water from the same storage tank throughout the whole experiment. The isotopic signature of irrigation water was repeatedly determined showing mean δ^*18*^ *O* of −8.79 ± 0.53 ‰ (*n* = 10).

**Table 1:** **List of species and additional information on plants and leaf anatomical traits**.

	**Origin**	**Leaf habit**	**Stomata**	**Life form**
*P. abies*	Boreal, Submeridional	Evergreen needle	Amphistomatous	Tree
*O. triangularis*	(Sub)tropics	Evergreen	Hypostomatous	Herb
*C. arabica*	Subtropics, Highlands	Evergreen	Hypostomatous	Shrub
*F. sylvatica*	Temperate	Deciduous	Hypostomatous	Tree
*A. longifolia*	Subtropics	Evergreen	Amphistomatous	Shrub
*Q. suber*	Mediterranean	Evergreen	Hypostomatous	Tree
*P. lanceolata*	Temperate	Evergreen perennial	Amphistomatous	Herb

### Measurements of leaf traits

Stomatal density (mm^−2^) and guard cell length (μm) were determined (*n* = 15) from stomatal imprints by randomly covering leaf surfaces of both leaf sides with a thin layer of transparent nail polish (Kardel et al., 2010). High resolution pictures of imprints were taken with a light microscope (Zeiss Alitplano, Oberkochen, Germany) connected to a camera (Sony Nex-5, Tokio, Japan). Stomates were counted and guard cell length measured. This method could not be applied to *Q. suber*, hence values for stomatal density and guard cell length were taken from literature (Molinas, 1991). Three to five leaves of each individual were taken to determine leaf water contents. For *P. abies* three times five needles (*n* = 3–5) were collected while five needles were treated as one sample.

### Measurements of oxygen isotopes and gas exchange parameters

#### Experimental design

Measurements of fluxes and oxygen isotope signatures of transpiration were conducted on one single leaf of three to five individuals of each species (*n* = 3–5). Young, fully expanded leaves at similar levels of irradiance were chosen for measurements. Leaves were exposed to two alterations in relative air humidity (*rH*) from 60 to 30% and back to 60%. Relative humidity was chosen as varying environmental parameter in this study, since it was shown to cause the strongest response in δ_*E*_ in previous studies (Simonin et al., 2013; Dubbert et al., 2014a). The response in δ_*E*_ and gas exchange parameters to these changes in *rH* was examined. Oxygen isotopic signatures (δ^*18*^ *O*) are reported as 60 s averages and gas exchange parameters were similarly recorded every 60 s. The enclosed leaves were exposed to climate chamber and, hence, plant growth conditions, with an *rH* of 60 % and a photosynthetic active radiation *(PAR)* of 400 μmol m^−2^ s^−1^, for at least 80 min (referred to initial). Subsequently, the enclosed leaves were exposed to two changes: (I) *rH* decreased from 60 to 30%; (II) *rH* increased from 30 to 60%. After each change in *rH*, the leaves were allowed to equilibrate for 120 min before the next change was induced. Over the whole experiment, reference gas and sample gas were measured alternately.

#### Coupling of leaf gas exchange system and laser spectrometer

Oxygen isotopic signatures of water vapor (δ^*18*^ *O*) were measured using Cavity Ring-Down Spectroscopy (CRDS) (L2120-i, Picarro, Santa Clara, CA, USA). Simultaneously, gas exchange parameters were collected using a portable gas exchange system (GFS-3000, Heinz Walz, Effeltrich, Germany). Environmental variables in the leaf cuvette were controlled by the gas exchange system (Figure S1) and set to 22°C leaf temperature at incident light at leaf surface of 400 μmol m^−2^ s^−1^. *rH* was reduced to 30% using the humidity control of the GFS 3000, while during the initial phase and during step change 2 “ambient” vapor of the climate chamber was used, which was permanently set to 60 %. Gas exchange parameters (transpiration rate *E* and stomatal conductance *g*_*s*_) were calculated based on von Caemmerer and Farquhar (1981).

Oxygen isotopic signatures of transpired water (δ_*E*_) were determined by mass balance (Dubbert et al., 2014a; Barbour et al., 2016):

(1) $$\begin{array}{l} \delta_{E}=\frac{u_{out}w_{out}\delta_{out}-u_{in}w_{in}\delta_{in}}{u_{out}w_{out}-u_{in}w_{in}}\\ =\frac{w_{out}\delta_{out}-w_{in}\delta_{in}}{w_{out}-w_{in}}-\frac{w_{in}w_{out}\left(\delta_{out}-\delta_{in}\right)}{w_{out}-w_{in}} \end{array}$$

where *u* is flow rate [mol (air) s^−1^], *w* is mole fraction [mol (H_2_O) mol (air)^−1^] and δ is isotope ratio of air, where subscripts denote the incoming (*in*) and outgoing (*out*) air stream of the chamber. δ, *w* and *u* were measured by alternately connecting the L2120-i with the sample and reference gas flow of the GFS-3000. Reference gas was measured for 10 min, sample gas measurements varied between 15 and 40 min depending on the progress of the measurement setup. The isotopic signature of the ingoing air stream was kept constant throughout the experiment, by choosing distilled water with matching isotopic composition for the humidifier of the GFS (mean values during the initial phase and following step changes 1 and 2 were, −13.4 ± 0.2, −13.6 ± 0.2, and −13.1 ± 0.3 ‰, respectively). During measurements the L2120-i was calibrated regularly using a standards delivery module and vaporizer (Picarro, Santa Clara, CA, USA) with two laboratory standards, which were calibrated against SLAP and VSMOW (IAEA, Vienna) before the experiment started.

#### Oxygen isotope signatures of leaf and soil samples

Bulk leaf, xylem, and soil samples (all *n* = 3–5) were collected at the end of experimental measurements. Leaf, xylem, and soil water was extracted on a custom build vacuum line by cryogenic distillation. Samples were heated at approximately 95°C for 90 min under vacuum of 0.8 Pa and vapor was trapped in liquid N_2_ cooled water traps. Samples were stored in sealed glass vials at 4°C until analysis. Water δ^*18*^ *O* was analyzed after headspace equilibration for 24 h at 20°C on an Isoprime IRMS (Elementar, Hanau, Germany) coupled via open split to a μgas auto sampler (Elementar, Hanau, Germany). Within every batch of 44 samples, three replicates of three different laboratory standards were analyzed for δ^*18*^ *O* calibration vs. V-SMOW. Laboratory standards were regularly calibrated against V-SMOW, SLAP, and GISP (IAEA,Vienna). Analytical precision was ~0.1‰.

### Isotope theory

The first to develop an equation describing isotopic fractionation associated with evaporation of water were Craig and Gordon (1965). Accordingly, the isotopic ratio of evaporation *R*_*E*_ is linked to the isotopic ratios of water at the evaporating sites *R*_*e*_ and ambient vapor *R*_*a*_ (Craig and Gordon, 1965):

(2) $$\begin{array}{l} R_{E}=\frac{1}{\alpha_{k}\alpha^{+}\left(1-h\right)}\left(R_{e}-\alpha^{+}hR_{a}\right) \end{array}$$

with α_*k*_ and α^+^ being the kinetic and equilibrium fractionation factors (>1), respectively and *h* the relative humidity corrected for leaf temperature (see Table 2 for abbreviations). The Craig and Gordon steady-state model requires that the isotopic composition of vapor departing from the leaf must be the same as the isotopic composition of incoming water: *R*_*E*_ = *R*_*s*_. This leads to:

(3) $$\begin{array}{l} R_{c}=\alpha_{k}\alpha^{+}\left(1-h\right)R_{s}+\alpha^{+}hR_{a} \end{array}$$

where *R*_*C*_ is the isotopic composition of leaf water at the evaporating site in steady state.

**Table 2:** **Used symbols and descriptions**.

**Symbols**	**Descriptions**
α_*k*_	Kinetic fractionation factor
α^+^	Equilibrium fractionation factor
δ^*18*^ *O*	Oxygen stable isotope signature (‰)
δ	Shortened for oxygen stable isotope signature (‰)
Δ	Deviation of a given isotopic signature from source water
℘	Péclet number
θ	Volumetric soil water content (m^3^ m^−3^)
*C*	The molar water concentration (mol m^−3^)
*D*/*D_*i*_*	Differences in molecular diffusivity (*D*) between the major and the minor isotopologue
*E*	Plant transpiration (mmol m^−2^ s^−1^)
*f*	Fractional difference between the leaf water enrichment of the bulk leaf (*Δ_m_*) and at the evaporating sites (*Δ_e_*)
*f_*1, 2*_*	Factors for estimating *R*_*l*_
*f_*em*_*	Factor for estimating *R_*m*_*
*g_*s*_*	Total conductance for water vapor
*h*	Relative humidity normalized to leaf temperature (%)
*L*	Effective length of water movement in the leaf mesophyll (m)
*n*	Exponent relating *D*/*D_*i*_* to apparent kinetic fractionation
*rh*	Relative air humidity (%)
*R*	Isotope ratio of (^18^O)/(^16^O)
*T*	Temperature (°C)
*u*	Flow rate (mol(air) s^−1^)
*W*	Leaf water volume (mol(H_2_O) m^−2^),
*w*	Mole fraction (mol(H_2_O) mol(air)^−1^)
τ	Leaf water time constant
**Subscripts**	**Descriptions**
*a*	Atmospheric air
*C*	Craig and Gordon steady-state prediction at the evaporating (‰)
*e*	Evaporating site
*e*(*t*)	Leaf-water at the evaporating sites at time *t* (‰)
*e*(*t*+*dt*)	Leaf-water at the evaporating sites at time plus a time step *t*+*dt* (‰)
*rH*	Relative humidity
*i*	Stomatal cavity
*in*	Chamber air
*l*	Leaf
*L*	Liquid bulk leaf water
*out*	Background air
*p*	Precipitation
*s/x*	Source water; xylem water

The non-steady-state isotopic composition of leaf water at the evaporating site *R*_*l*_ can be written in an iterative form, if leaf water volume *W* [mol (H_2_O) m^−2^] is assumed constant (Dongmann et al., 1974; Farquhar and Cernusak, 2005; Cuntz et al., 2007):

(4) $$\begin{array}{l} R_{l}\left(t+dt\right)=R_{C}+\left(R_{l}\left(t\right)-R_{C}\right)e^{-\frac{dt}{\tau }} \end{array}$$

where *R*_*l*_ at a time *t* + *dt* is calculated from *R*_*l*_ at an earlier time *t* with constant environmental conditions during the time step *dt*. *g*_*s*_ is the leaf conductance for water vapor from the stomatal cavity to the point of observation, and *w*_*i*_ the humidity in the stomatal cavity, i.e., vapor saturation at leaf temperature expressed as mole fraction [mol(H_2_O) mol(air)^−1^].

Following Farquhar and Cernusak (2005) the time constant (τ), can be described as:

(5) $$\begin{array}{l} \tau =\left(1-f\right)\frac{W}{g_{s}w_{i}}\alpha_{k}\alpha^{+} \end{array}$$

with α_*k*_ and α^+^ ≈ 1:

(6) $$\begin{array}{l} \tau =\left(1-f\right)\frac{W}{g_{s}w_{i}} \end{array}$$

Recently, Song et al. (2015) suggested a modified formulation for τ specifically suitable for modeling isotopic signatures of leaf water and transpirative fluxes in a cuvette environment, substituting *g*_*s*_ × *w*_*i*_ with *E* and accounting for the proportional difference between *w*_*in*_ and *w*_*i*_. In a cuvette scenario with the ingoing airstream (*w*_*in*_) often being dry air (as in Song et al., 2015), this effectively corrects for the strong influence of *E* on isotopic signatures of vapor (δ_a_).

This leads to:

(7) $$\begin{array}{l} \tau =\left(1-f\right)\frac{W}{E}\left(1-\frac{w_{in}}{w_{i}}\right) \end{array}$$

We used this updated formulation for cuvette scenarios to calculate δ_*E*_ and hence also τ.

The factor *f* in Equation (5–7) denotes a Péclet term, with

(8) $$\begin{array}{l} f=\frac{1-e^{-{℘}_{m}}}{{℘}_{m}}\;with\;the\;Péclet\;number\;{℘}_{m}=\frac{EL}{CD} \end{array}$$

where *C* = 10^6^/18 = 55.6·10^3^ mol m^−3^ is the molar water concentration, *D* (m^2^ s^−1^) is the tracer diffusivity in liquid water and *L* (m) is the effective length of water movement in the leaf mesophyll. *E* is the transpiration rate in mol m^−2^ s^−1^.

The isoflux of transpiration is expressed as the product of *E* and δ_*E*_. In this experiment we calculated the isoflux as the product of *E* and Δ_*E*_, as our main interest was in the impact of non-steady-state δ_*E*_ (Δ_*E*_ = δ_*E*_ − δ_*X*_).

### Statistical analysis

Kruskal-Wallis tests were used to test for species specific differences in stomatal density, size, leaf water content, E, g_*s*_, and τ. The same test was used to test for species-specific differences in the magnitude of decrease/increase in the isotopic signature of transpiration, τ, and mean isofluxes of the three experimental stages in response to changes in *rH*. We performed a *t*-test to compare the isotopic signatures of transpiration with that of xylem water at the initial phase of the experiment. Non-linear correlations were used to relate stomatal conductance with stomatal density and guard cell length, stomatal density to guard cell length and leaf water content with stomatal traits. Power functions were used to relate species-specific differences in τ and isofluxes with their differences in g_*s*_/W and stomatal density/W. In case significant relationships could be detected (*p* < 0.05), *R*^2^ and *p*-values are given. Statistical analyses were carried out with the software STATISTICA 6.0 (StatSoft Inc., Tulsa, OK, USA).

## Results

### Leaf traits and transpiration

**Figure 1:**
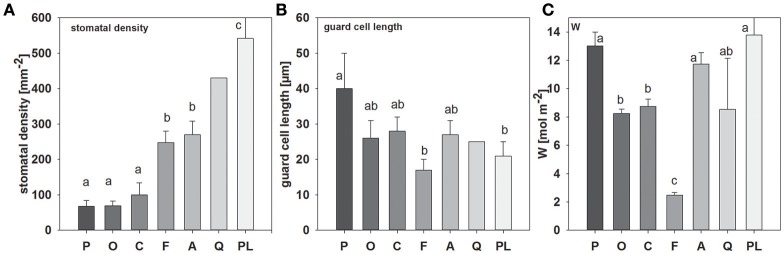
**Mean values (±SD, ***n*** = 3–5) of (A)** stomatal density, **(B)** guard cell length and **(C)** leaf water content (W), species-specific gray scale color code is maintained throughout all figures (except Figure 3). Species are sorted as follows: *P*. *abies, O. triangularis, C. arabica, F. sylvatica, A. longifolia, Q. suber, P. lanceolata*. Lower case letters indicate statistical differences between species (*p* < 0.05).

Significant differences in stomatal density and guard cell length could be observed between species (Figure 1; see also Figure S2). *Picea abies* and *O. triangularis* had lowest stomatal densities of 68 ± 16 and 69 ± 14 mm^−2^, respectively. Highest stomatal densities were observed in *P. lanceolata*, with 542 ± 86 mm^−2^. Notably, three of the studied species, *P. abies, A. longifolia*, and *P. lanceolata*, possess amphistomatous leaves, hence stomatal densities are expressed here as total stomatal densities of both leaf sides. In addition, differences in guard cell length could be found, with a tendency toward smaller guard cell length at higher stomatal density (*R*^2^ = 0.44; *p* = 0.08; ranging between 17 ± 3 μm in *F. sylvatica* and 40 ± 10 μm in *P. abies*). Moreover, together stomatal density and guard cell length (i.e., stomatal density ^*^ guard cell length) explained species-specific differences in stomatal conductance quite well (*R*^2^ = 0.96, *p* < 0.01). Leaf water content (*W*) also differed considerably between species, but differences were not related to stomatal traits (Figure 1). Three species contained high leaf water contents between 11.7 ± 0.8 and 13.8 ± 2.8 mol m^−2^ (*P. abies, A. longifolia, P lanceolata*), while medium leaf water contents could be found in *O. triangularis, C. arabica*, and *Q. suber* (~8.5 mol m^−2^). *Fagus sylvatica* had by far the lowest leaf water content of 2.5 ± 0.2 mol m^−2^.

**Figure 2:**
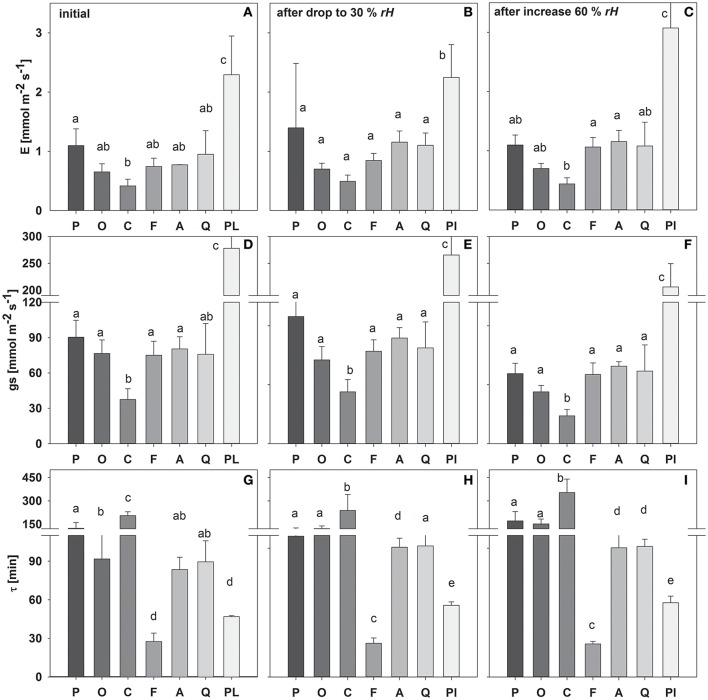
**Mean transpiration rate (***E***), stomatal conductance (***g***_**s**_) and time constant (τ) during the initial experimental phase (A,D,G)**, following a drop in rH **(B,E,H)** and an increase in rH **(C,F,I)**. Species are sorted as follows: *P*. *abies, O. triangularis, C. arabica, F. sylvatica, A. longifolia, Q. suber, P. lanceolata*. Lower case letters indicate statistical differences between species (*p* < 0.05).

During the first initial stage of the measurement period, most species exhibited transpiration rates and stomatal conductance of around 0.6–1.2 and 70–90 mmol m^−2^ s^−1^, respectively (Figure 2). Only *C. arabica* and *P. lanceolata* showed significantly smaller (*E* = 0.41 and *g*_*s*_ = 37 mmol m^−2^ s^−1^) and higher (2.3 and 278 mmol m^−2^ s^−1^) *E* and *g*_*s*_, respectively.

Similarly, the leaf water time constant τ (i.e., Equation 8) in the initial experimental phase varied between species, which accordingly can be divided into 3 main groups (Figure 2): (I) τ < 50 min (*P. lanceolata and F. sylvatica*); (II) τ > 50 but < 100 min (*A. longifolia, Q. suber, O. triangularis*) and III) τ > 100 min (*C. arabica, P. abies*). It has to be noted that *O. triangularis* faced a significant increase in τ following the first change in *rH*, thereafter belonging to group III (Figure 2). Mean values of measured bulk and modeled evaporating site leaf isotopic signatures is given in Table S1.

### Oxygen isotope signatures of transpired vapor and the transpiration isoflux

**Figure 3:**
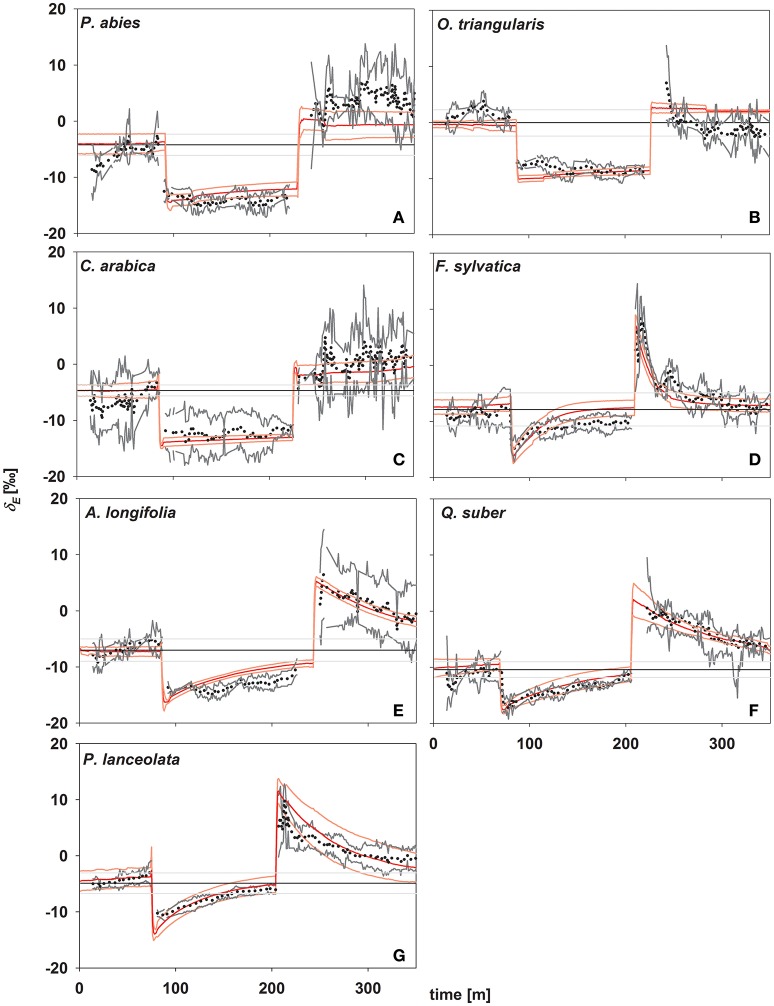
**Time series of oxygen isotope signatures of transpired water vapor estimated by laser spectroscopy (in ‰; black dots; gray lines denote standard deviation; ***n*** = 3–5) and modeled (red lines, light red lines denote standard deviations; ***n*** = 3–5)**. Black lines denote source water for plant transpiration (mean values ± SD in ‰, *n* = 3–5). **(A)** *Picea abies*; **(B)** *Oxalis triangularis*; **(C)** *Coffea arabica*; **(D)** *Fagus sylvatica*; **(E)** *Acacia longifolia*; **(F)** *Quercus suber*; **(G)** *Plantago lanceolata*.

In concert with transpiration, isotopic signatures of leaf transpiration (δ_*E*_) were measured first at environmental conditions of the climate chamber (*rH* = 60%), and following a decrease (to 30%) and increase (to 60%) in *rH* (Figure 3). In addition, δ_*E*_ was modeled using Equations (2) and (4), using the formulation for the time constant (Equation 7) as described by Song et al. (2015). During the initial stage, plants transpired at isotopic steady state, i.e., no significant deviation from source water isotopic signature (Figure 3) and a high agreement between measured and modeled isotopic signatures of transpiration (δ_*E*_) was found. This can be expected here, as all plants were kept under stable environmental conditions regarding temperature and relative humidity within the climate chamber environment at all times. Further, during the initial phase of the experiment the conditions of the air entering the cuvette matched those within the climate chamber.

In response to environmental changes, (i.e., decreasing and increasing *rH*) a direct strong decrease/increase in δ_*E*_ was observed in all species. Notably, the magnitude of this immediate response of δ_*E*_ directly following a change in *rH* did not differ between species and was −8.27 ± 0.67 and 11.35 ± 0.9‰, respectively after decrease and increase in *rH*. In contrast, the studied species showed different behavior in the subsequent approach to isotopic steady state and three distinct groups could be identified, in accordance to their time constant. Only species of group I (*P. lanceolata* and *F. sylvatica*) reached isotopic-steady-state transpiration after 120 min of stable environmental conditions. Group III *(P. abies, O. triangularis*, and *C. arabica)* did not reach isotopic steady state even after 120 min of unchanging environmental conditions, while group II *(A. longifolia* and *Q. suber)* approached steady state but did not fully reach it (Figure 3).

**Figure 4:**
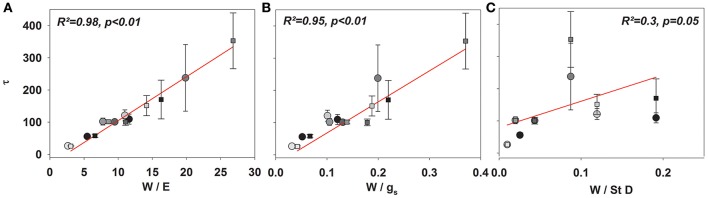
**Mean τ (Equation 8) across all species subsequent to a drop to 30% (circles) and an increase to 60% ***rH*** (squares) against ***W/E*** (A)**, *W/g*_*s*_ **(B)**, and *W/*stomatal density **(C)**.

Given the good agreement between modeling results and observations, the time constant (Equations 2, 4, and 7) described the approach of δ_*E*_ to isotopic steady state following environmental perturbation rather well (Figure 3). Species-specific differences in the time constant (τ) could be very well described by the term W/E and W/g_*s*_ (*R*^2^ = 0.98 and 0.95; *p* < 0.01, Figures 4A,B). Moreover, although showing a weaker relationship, species specific differences in τ were also significantly related to the term W/stomatal density (providing a rough estimate of g_max_; *R*^2^ = 0.3, *p* < 0.05, Figure 4C).

In addition to the quantification of the isotopic non-steady-state effect of δ_*E*_ in response to changes in *rH*, the isoflux of the transpired vapor was determined for all three stages of the experiment. Here, transpiration isofluxes were calculated relative to xylem/source water δ^*18*^ *O* because despite equal irrigation source for all species, averaged soil water δ^*18*^ *O* differed between species, most probably due to evaporative enrichment of soils without dense soil cover, leading to different water residence times in the soil (data not shown).

**Figure 5:**
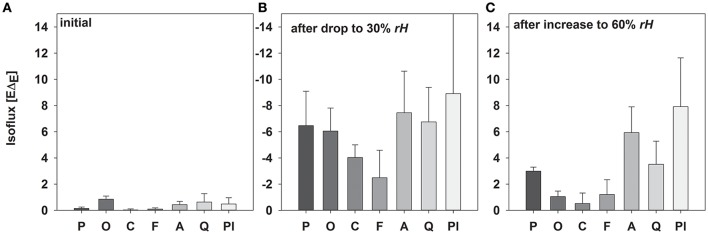
**Mean values (±SD, ***n*** = 3–5) of isofluxes (−***E***Δ_***E***_; mean values ± SD, ***n*** = 3–5) during the initial experimental setup (A)**, after reducing *rH* **(B)** and increasing *rH* **(C)** of *Picea abies, Oxalis triangularis, Coffea arabica, Fagus sylvatica, Acacia longifolia, Quercus suber, Plantago lanceolata*. The entire time between step changes was used for calculations.

**Figure 6:**
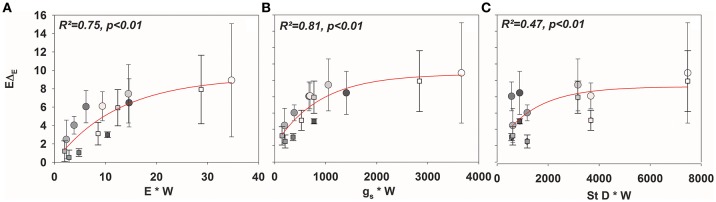
**Mean values (±SD) of isofluxes across all species (***E***Δ_***E***_; mean values ± SD, ***n*** = 3–5) against (A)** E × W, **(B)** *g*_*s*_× *W*, and **(C)** stomatal density × *W*.

**Figure 7:**
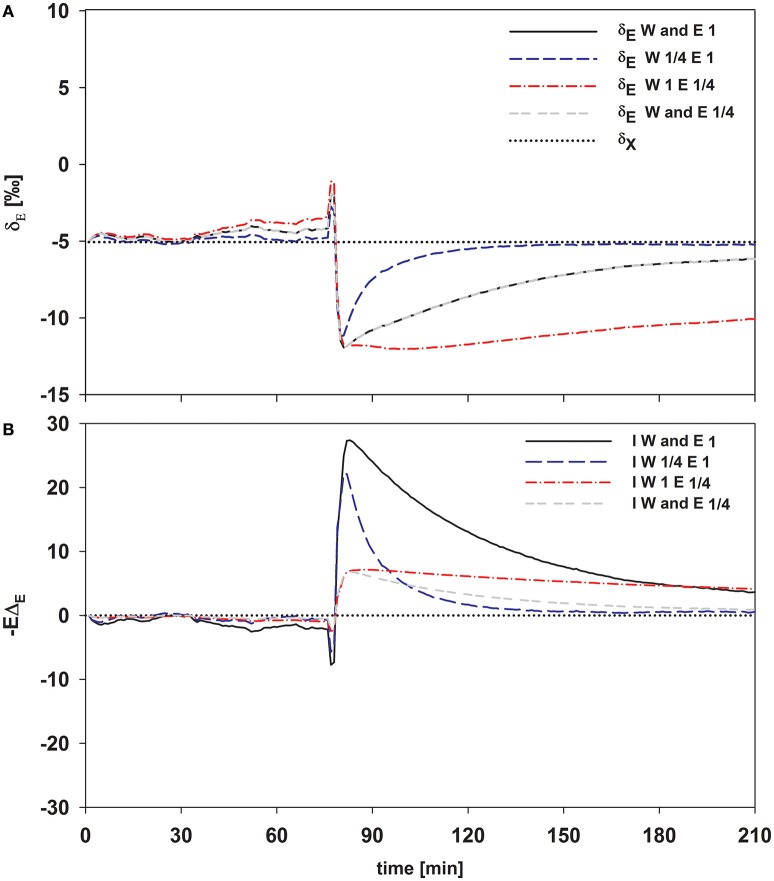
**Time series of modeled δ_***E***_ (A)** and the transpirational isoflux **(B)** of *Plantago lanceolata*. To disentangle the temporal dynamics of the impact of *W* and transpiration rate, *E* was set to one and 1/4 of the observed value and *W* was set to 14.4 and 3.6 mol m^−2^ (which is 1/4 of 14.4). These values span the values for *E* and *W* observed in this study pooled over all species.

As expected, isofluxes were around zero during the initial stage, as plants were transpiring in or near to isotopic steady state (i.e., Δ_*E*_ ≈ 0; Figure 5A). Following step changes, mean isofluxes over the measurement duration after changes in *rH* differed between species (Figures 5B,C), varying between 3.2 and 12.9‰ mmol m^−2^ s^−1^ after the drop in *rH* and between 0.8 and 8.8‰ mmol m^−2^ s^−1^ after increasing *rH*. Again, significant relationships were found between mean isofluxes during the second and third experimental stage and the terms E × W, g_*s*_ × W and stomatal density × W (Figure 6). Finally, we modeled the temporal development of the transpirational isoflux of *Plantago lanceolata* assuming 2 different values for *E* and *W*. Original values of *E* and *W* of *P. lanceolata* were therefore divided by 4 (Figure 7), representing the range for *E* and *W* observed in this study across all species (Figures 1, 2). The impact of changing E and *W* on δ_*E*_ (Figure 7A) are as can be expected from theory (i.e., Equations 2 and 4). However, the impact of changes in *E* and *W* on the transpirational isoflux is strongly depending on the time of observation/integration (Figure 7B). Up to 10 min following the drop in *rH*, the smallest isoflux can be observed for the model run with small *E* and the run with small *E* and *W* (on average 6.3 and 6.1‰ mmol^−2^ m^−2^ s^−1^ compared to 21.5‰ mmol^−2^ m^−2^ s^−1^ considering high *E* and *W*, see also Table 3). However, the longer the time of integration is, the higher becomes the relative influence of *W*. Integrating over the full 120 min before the next step change, mean isofluxes were smallest considering small *E* and small W and *E* (2.3 and 2.6 ‰ mmol^−2^ m^−2^ s^−1^, respectively, see Table 3).

**Table 3:** **Mean isoflux integrated over 10, 30, 60, and 120 min following a decrease in ***rH*** considering differences in W and ***g***_***s***_**.

**Isoflux**	**10 min**	**30 min**	**60 min**	**120 min**
W and g_*s*_ 1	24.1 ± 4.4	21.5 ± 3.8	16.9 ± 5.5	10.7 ± 6.8
W 1/4 g_*s*_ 1	16.9 ± 3.8	9.7 ± 5.9	5.6 ± 5.9	2.3 ± 4.6
W 1 g_*s*_ 1/4	6.3 ± 1.4	6.6 ± 0.9	6.3 ± 0.7	5.4 ± 0.9
W and g_*s*_ 1/4	6.1 ± 1.1	5.4 ± 0.9	4.2 ± 1.4	2.6 ± 1.7

*1, observed g_s_ and W; 1/4, quarter of observed g_s_ and W*.

## Discussion

### Non-steady state effect of δ_*E*_ and the relation to leaf traits

Oxygen isotope signatures of transpiration are increasingly used to partition evapotranspiration differing at spatial (plant to global) and temporal (minute to annual) scales. The present results emphasize that assuming plant transpiration to be in isotopic steady state can have a large impact i.e. for ecosystem partitioning studies (Yakir and Sternberg, 2000; Yepez et al., 2003; Williams et al., 2004; Zhang et al., 2011; Dubbert et al., 2013, 2014b; Hu et al., 2014). Current approaches modeling the isotopic signature of leaf water allowing for isotopic non-steady state are often based on the assumptions of one unenriched water pool in a leaf (i.e., vein water) and enrichment occurring at the evaporative sites within the mesophyll. They predict that the time needed for the isotopic signature of leaf water at the evaporating sites (δ_*e*_) to approach isotopic steady state under stable environmental conditions is dependent on the time constant (τ; Dongmann et al., 1974; Farquhar and Cernusak, 2005; Cuntz et al., 2007). The deviation of the isotopic signature of transpired vapor from steady state (Δ_*E*_) is tightly linked to Δ_*e*_, however deviations of δ_*e*_ from isotopic steady state are amplified dependent on relative humidity (Δ_*E*_ = (Δ_*e*_ − Δ_*C*_)/($\alpha^{+}\alpha_{\text{k}}$(1 − *h*)); see Dubbert et al., 2014a). Consequently, analyzing the temporal development of δ_*E*_ and its deviation from isotopic steady-state (Δ_*E*_), one needs to separate environmental from leaf trait related drivers, *g*_*s*_ (*E* under cuvette scenarios) and W that are influencing δ_*E*_ via the leaf water time constant term in equation 4, i.e., δ_*e*_.

Responses to changes in *rH* were analyzed regarding two distinct aspects: the immediate response following a step-change in *rH* and the subsequent approach to isotopic steady state under stable environmental conditions. While changes in *rH* directly influence δ_*E*_, changes in other environmental parameters like variations in PPFD or *c*_*a*_, as analyzed in previous studies (Simonin et al., 2013), indirectly influence δ_*E*_ via their control on *g*_*s*_ and *E*. Notably, the magnitude of the direct response of δ_*E*_ to step changes did not differ between species. Considering Δ_*E*_ to be the deviation of Δ_*e*_ from Δ_*C*_ amplified by 1-*h*, it becomes apparent the that the magnitude of the immediate response after a step change is directly related to the magnitude of the step-change in rH and its influence on 1-*h* as well as δ_*C*_ (see Equation 3).

In contrast, we found significant differences in the approach to isotopic steady state subsequent to changes in *rH*. Notably, the Dongmann-style model (see Equation 4) as used here predicted the observed temporal dynamics in δ_*E*_ following step-changes in rH reasonably well (Figure 3; see also Simonin et al., 2013). We can clearly see that τ can be nicely predicted by E or g_*s*_ and leaf water content (Farquhar and Cernusak, 2005; Song et al., 2015). Interestingly, neither of these two parameters alone was able to predict changes in τ and hence non-steady-state δ_*E*_ pooled across all species. The species-specific reaction to the decrease of *rH* led to significant differences in *E* and hence τ before and after changes in *rH*. Particularly *P. abies and O*. triangularis strongly decreased *g*_*s*_ and hence *E* following the step change from 60 to 30% *rH*. Therefore, the responsiveness of the stomatal aperture to changing environmental conditions seems to be an importing factor characterizing τ and thus non-steady-state effects of δ_*E*_. However, the still significant relationship between τ and *W*/stomatal density, seem worthwhile for further investigation, potentially providing very easy to quantify means for a priory assumptions of the impact of isotopic non-steady state in ecosystem with distinct plant functional groups. This could be beneficial especially for approaches aiming at partitioning evapotranspiration by use of stable isotopes. We believe that the possible impact of assuming transpiration to be in isotopic steady state on the final partitioning approach could be thus estimated a priori.

Song et al. (2015) demonstrated that in a cuvette scenario, τ *is* not influenced by the gross flux of water (g_*s*_× w_*i*_) but rather by the net flux of water from the leaf (*E*) as well as the impact of *E* on cuvette vapor. Consequently they adapted the model of τ by Farquhar and Cernusak (2005). This “leaf cuvette effect” leads to higher τ values than expected for open field scenarios. In fact, with w_*in*_ being dry air, Song et al. (2015) showed that τ in a cuvette is twice as high as under field conditions. While this effect will be smaller when w_*in*_ is not dry air as in this study (see Figure S3), it is still very important to consider this effect in studies involving cuvette-based measurements. Moreover, it should also be kept in mind that τ under field conditions will be smaller and hence the total impact of non-steady-state transpiration following changes in environmental conditions will be somewhat lower than predicted by cuvette measurements.

### Isofluxes and impact on isotopic signatures of atmospheric vapor

Oxygen isotope signatures of transpiration are used to trace water flows through ecosystems, but also to assess the impact of plant transpiration on atmospheric water vapor (Xiao et al., 2010, 2012; Lee et al., 2012). In this regard, it is important to understand the impact of environmental and plant physiological controls on the impact of isotopic non-steady-state effects of δ_*E*_ on atmospheric vapor. The impact of isotopic non-steady-state δ_*E*_ on atmospheric vapor (isoforcing), however, is not only dependent on the δ_*E*_ signature, but on the product of E and δ_*E*_ (i.e., the isoflux of the transpired vapor). Furthermore, the actual isoforcing (Lee et al., 2009) of transpired vapor under natural conditions will also be influenced by changes in the H_2_O concentration and isotopic signature of atmospheric vapor.

Dubbert et al. (2014a) showed that under natural field conditions the transpirational isoforcing of Mediterranean *Q. suber* trees significantly deviated from the steady-state assumption at the least on short time scales (i.e., less than 24 h). In this experimental study, we calculated isofluxes as a deviation from the steady-state isoflux (i.e., –*E*Δ_*E*_). Isofluxes of transpired vapor strongly differed between species and differed significantly from isotopic steady-state isofluxes after changes in *rH*, highlighting the potential forcing of isotopic non-steady-state transpiration on atmospheric vapor (Figure 6). Previous studies suggested, that isotopic non-steady-state effects of leaf transpiration will have a significant impact on atmospheric vapor, when the time constant (τ) is long, i.e., low *E* (or g_*s*_ in non-cuvette scenarios) and/or high *W* (Lai et al., 2006; Simonin et al., 2013; Dubbert et al., 2014a). Here, species-specific differences in τ and hence in δ_*E*_ can originate from either differences in *E* or *W*, while the observed differences in transpiration rate (*E*) are not influenced by *W*. The impact of *W* on the non-steady-state transpirational isoflux can be clearly predicted: high *W* will lead to a higher time constant and hence to a slower return to isotopic steady-state transpiration. This is more complex for *E* because higher *E* will lead to a smaller time constant and hence a faster return to isotopic steady-state transpiration, but also directly increases the term −*E*Δ_*E*_ (under a given *rH*).

Figure 7 clearly shows that within the first 10–30 min following a step change the direct impact of *E* on −*E*Δ_*E*_ dominates the development of the isotopic non-steady-state isoflux. Differences in *W* and its influence on τ and hence δ_*E*_ are getting increasingly important though if the time of integration becomes greater than 30 min (Figure 7B). Hence, if variation in the time constant is driven by changes in *E*, it has a bigger short-term effect on the resulting isoflux than if the source of variation is *W*, due to the direct effect of *E* on −*E*Δ_*E*_. Similarly, a small time constant given by high *E* and high *W* results in a larger isoflux than a large time constant achieved via large *W* and small *E*, at least for time intervals from 10 to 30 min following environmental perturbation. Only in case of longer time periods with stable environmental conditions (>60 min), changes in *W* do become increasingly important for the non-steady-state isoflux, and it is questionable whether they are relevant to address under natural conditions.

Concluding, our results demonstrate the usefulness of coupled gas-exchange laser spectrometer set-ups to analyze water isotope fractionation processes at the leaf scale. The overall very good agreement between observations and modeling results in seven species with distinct leaf traits supports current modeling approaches regarding leaf water isotopic enrichment. Our results provided for the first time a species survey on the impact of distinct leaf traits on the temporal dynamics of the deviation of δ_*E*_ from isotopic steady state. We demonstrate that isoforcing of δ_*E*_ on atmospheric vapor is strongly driven by control of *E* over −*E*Δ_*E*_, even though high *E* also leads to small time constants. By contrast, impact of *W* on the time constant only influences the non-steady-state isoflux on time scales probably not relevant under natural conditions. The significant relationships between *E, g*_*s*_, and even stomatal density (as a measure of *g*_max_) and *W* on the one hand and τ and the isotopic non-steady-state isoflux on the other hand are quite promising. Particularly morphological leaf traits are relatively easy to sample and measure under field conditions, and their use as a priori information of τ and isoflux deviation from the steady-state assumption would be highly useful. Transpirational isotopes provide the basis for environmental tracer/paleo-climatic studies and are used as tracers from leaf to atmospheric scale. Hence, our findings have wide reaching consequences across large temporal and spatial scales.

## Author contributions

MD and CW conceived the study design and planned the experiment. MD analyzed the data and wrote 90% of the manuscript, AK conducted the experiment and assisted with manuscript writing. CW commented on the manuscript. MD and AK equally contributed to this work.

## Funding

This work was funded by the German Science Foundation (DFG, #WE2681). The article processing charge was funded by the German Research Foundation (DFG) and the University of Freiburg in the funding programme Open Access Publishing. Grant number 2100095601, “Innovationsfond Forschung”, granted to MD.

### Conflict of interest statement

The authors declare that the research was conducted in the absence of any commercial or financial relationships that could be construed as a potential conflict of interest.
