# Supplementary material for: Impact of Leaf Traits on Temporal Dynamics of Transpired Oxygen Isotope Signatures and Its Impact on Atmospheric Vapor
Source: Front Plant Sci. 2017 Jan 18;8:5. doi: 10.3389/fpls.2017.00005 (PMC5241305; doi:10.3389/fpls.2017.00005)
Supplement: Supplementary file 1 [file DataSheet1.DOCX]

Supporting material


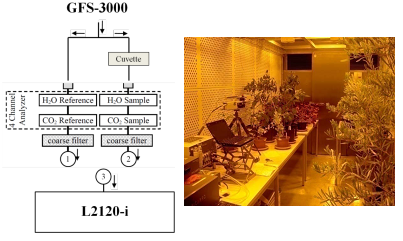


**Figure S1.** Left: The L2120-i was coupled with the GFS-3000 by isotope inert tubes leading from the GFS-3000 outlets "ANALYZER / SAMPLE" (1) and "ANALYZER / REFERENCE" (2) to the L2120-i inlet (3) (modified after Heinz Walz (2013)). Right: Climate chamber and measurement setup.


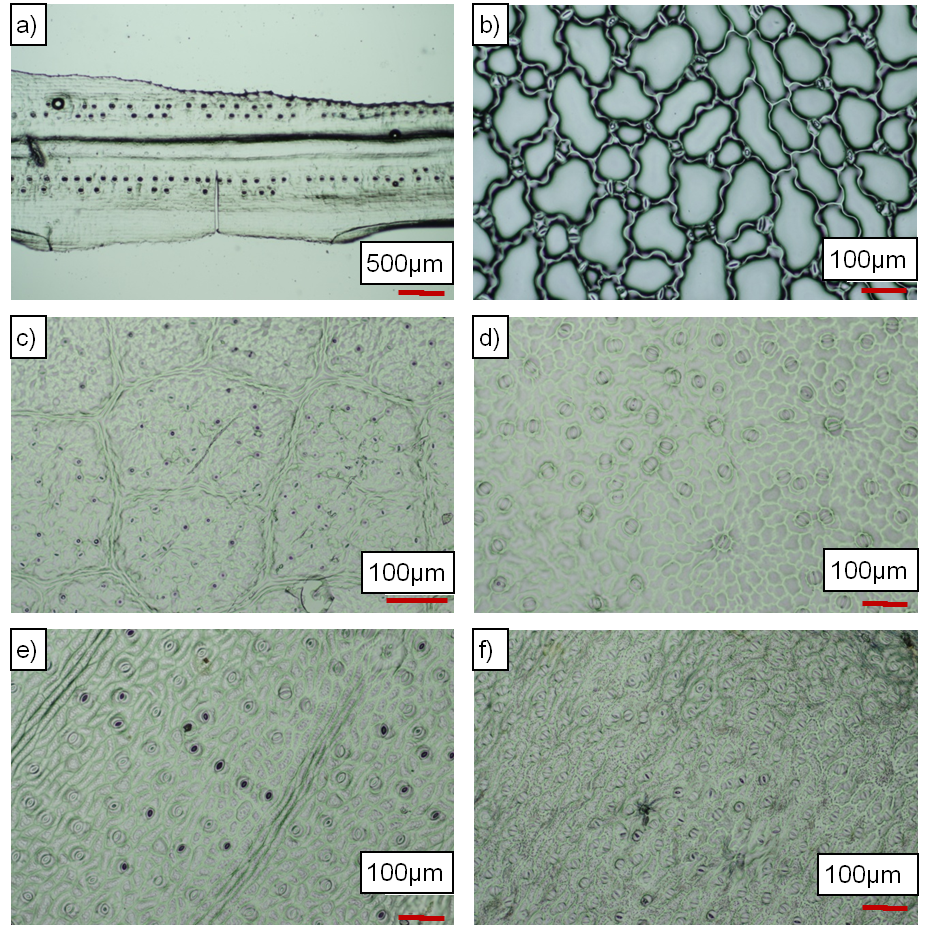


**Figure S2:** High resolution images of abaxial leaf surfaces of a) *Picea abies*; b) *Oxalis triangularis*; c) *Coffea arabica*; d) *Fagus sylvatica*; e) *Acacia longifolia*; f) *Plantago lanceolata.*


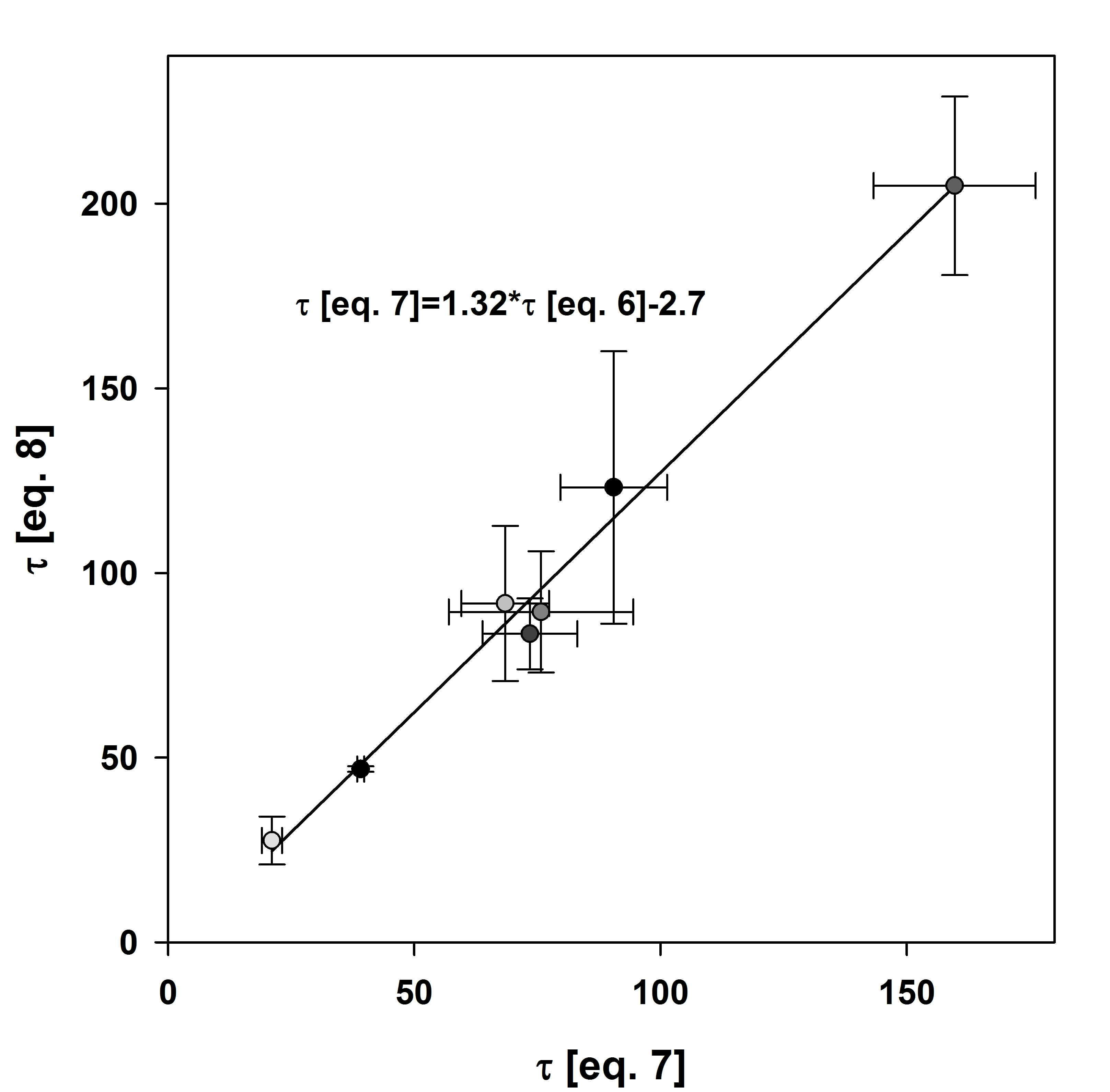


**Figure S3:** Leaf water time constant (τ) calculated using equation 7 versus τ calculated with equation 8. Values denote averages of each species prior to step changes. The black line denotes the linear regression with R²=0.99 and p<0.001.

**Table S1:** Mean values (±SD, n=3-5) of the isotopic enrichment of bulk leaf water relative to source water (*Δ*_L_) and the isotopic enrichment of leaf water at the evaporating site relative to source water (*Δ*_e_) for all studies species at the end of the experimental period at *rH* of 60 %.

|  | ***ΔL*** | ***±*** | ***Δe*** | ***±*** |
| --- | --- | --- | --- | --- |
| ***P. abies*** | 16.01 | 1.59 | 18.76 | 1.19 |
| ***O. triangularis*** | 16.37 | 1.46 | 18.94 | 1.20 |
| ***C. arrabica*** | 13.59 | 1.37 | 18.76 | 0.61 |
| ***F. sylvatica*** | 14.73 | 1.96 | 17.81 | 0.63 |
| ***A. longifolia*** | 9.03 | 0.01 | 11.96 | 0.00 |
| ***Q. suber*** | 17.71 | 1.45 | 20.64 | 1.71 |
| ***P. lanceolata*** | 6.18 | 1.77 | 9.90 | 2.23 |
